# Reemergence of Dengue in Mauritius

**DOI:** 10.3201/eid1604.091582

**Published:** 2010-04

**Authors:** Mohammad I. Issack, Vidula N. Pursem, Timothy M.S. Barkham, Lee-Ching Ng, Masafumi Inoue, Shyam S. Manraj

**Affiliations:** Central Health Laboratory, Candos Mauritius (M.I. Issack, V.N. Pursem, S.S. Manraj); Tan Tock Seng Hospital, Singapore (T.M.S. Barkham); Environmental Health Institute, Singapore (L.-C. Ng); Institute of Molecular and Cell Biology, Singapore (M. Inoue)

**Keywords:** Dengue, Mauritius, reemergence, PCR, viruses, dispatch

## Abstract

Dengue reemerged in Mauritius in 2009 after an absence of >30 years, and >200 cases were confirmed serologically. Molecular studies showed that the outbreak was caused by dengue virus type 2. Phylogenetic analysis of the envelope gene identified 2 clades of the virus. No case of hemorrhagic fever was recorded.

Mauritius is a tropical island nation of 1,865 km^2^ in the southwestern Indian Ocean, ≈2,000 km off the coast of eastern Africa. It has a population of 1.25 million, with ≈68% of Indian origin and 27% of predominantly African or mixed ancestry. It is classified by the World Bank as an upper-middle-income country. The economy is diversified; textiles, tourism, sugar cane, banking, and business process outsourcing are the main sectors. Most Mauritians have a sedentary lifestyle.

In 2008, a total of 930,000 visitors traveled to Mauritius for tourism and business; 98% arrived by air (*1*). They were mostly from western Europe (60.5%), Réunion island (10.3%), South Africa (9.1%), and India (4.7%).

Mauritius was, for many years, essentially free from indigenous dengue and chikungunya disease until 2005 and 2006 when outbreaks of chikungunya occurred, with *Aedes albopictus* mosquitoes as the vector (*2*). In the region, a well-documented epidemic of dengue fever caused by dengue virus type 2 (DENV-2) occurred on Réunion island in 1977–1978, and 2 outbreaks were caused by the same dengue serotype in the Seychelles in 1976–1977 and 1978–1979 (*3*,*4*). Although no record was made of laboratory-confirmed cases in the 1970s in Mauritius, a subsequent seroepidemiologic study suggests that cases of dengue also occurred in the country around that time, and it is reasonable to postulate that they were also caused by DENV-2 (*5*). Since then, apart from the occasional imported case, no evidence of dengue transmission has been reported for >30 years in Mauritius. However, dengue fever reemerged in the country in 2009, and we report on the laboratory investigation of the outbreak.

## The Outbreak

At the end of April 2009, a physician from a private clinic in Port-Louis, the capital city, reported having seen several patients with fever, malaise, diarrhea, increased levels of liver enzymes, and marked thrombocytopenia. Rash and arthralgia were not mentioned, and no test was requested for dengue or chikungunya viruses. On June 1, another physician requested dengue serologic testing for a patient who had fever for the past 10 days, rigors, generalized aches and pains, and a petechial rash. The patient had lived in Malaysia several years previously but had no history of recent travel. His liver enzyme levels were increased, and his thrombocyte count was 15,000 cells/μL. At the Central Health Laboratory (CHL) in Mauritius, the patient’s serum was positive for immunoglobulin (Ig) G and IgM against dengue with the Hexagon Dengue rapid immunochromatography test (Human GmbH, Wiesbaden, Germany) and negative for Platelia dengue nonstructural protein 1 (Bio-Rad Laboratories, Marnes-la-Coquette, France).

The next day, 6 patients who had previously been admitted with fever at the above-mentioned private clinic were traced by the Department of Public Health of the Ministry of Health and quality of Life (MoHQL). They all lived in the same suburb of the capital city. Serum samples from all 6 patients showed IgG and IgM against dengue. The MoHqL immediately initiated an action plan which included mosquito control measures by fogging and larviciding, environmental cleaning, and a public awareness campaign on how to eliminate mosquito breeding sites. The first 10 positive serum specimens, all of which had dengue antibodies were sent for confirmation to the National Health Laboratory Services in South Africa, where all were subsequently found to be positive for dengue IgG and IgM by hemagglutination-inhibition test and ELISA, respectively.

On June 5, dengue NS1 antigen was detected at CHL from serum specimens from 3 patients. Four days later, serum samples from 11 patients that were positive for dengue antigen were sent to Tan Tock Seng Hospital in Singapore for reverse transcription–PCR (RT-PCR) testing. Approximately 24 hours later, the laboratory reported that DENV RNA was detected in 7 of the samples by real-time RT-PCR, with previously described primers using SYBR Green and gel electrophoresis (*6*). The serum samples were later found to be positive for DENV-2 by multiplex RT-PCR with serotype-specific primers, and detection with serotype-specific probes by using a Luminex xMAP-based assay (Luminex, Austin, TX, USA) (unpub. technique).

Subsequently, nucleotide sequencing and phylogenetic analysis of the envelope gene from the PCR products of the 7 positive serum specimens showed that, although all the viruses belonged to the Cosmopolitan genotype, 2 separate clades were present. Four samples clustered with isolates from India, and the remaining 3 were most closely related to an isolate from Sri Lanka (Figure). Overall, during June, dengue NS1 antigen was detected in the serum specimens of 194 patients. In 40 other cases, the serum specimens tested positive for dengue IgM by immunochromatography or capture ELISA (Panbio, Brisbane, Queensland, Australia) but negative for NS1 antigen. All patients were clustered in suburbs of Port-Louis or had traveled there. Only 5 and 3 new cases of dengue were diagnosed in July and August, respectively, and no case was reported in September. The case-patients ranged in age from1 to 91 years; median age was 36 years, and 52.5% were male.

**Figure:**
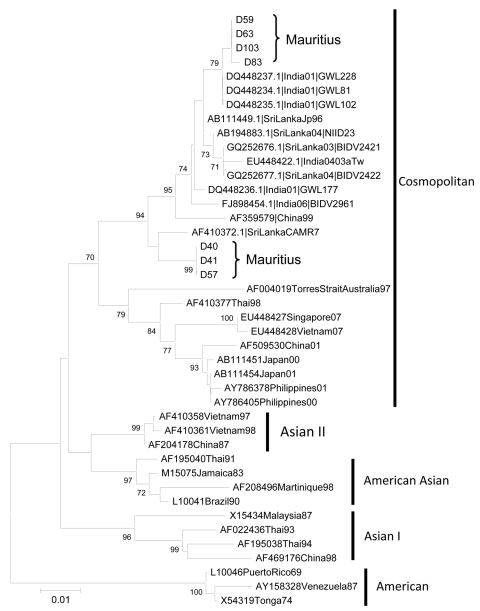
Phylogenetic relationships of dengue virus isolates from Mauritius inferred by envelope (E) gene sequence by using the maximum likelihood method as implemented in PAUP* version 4.0b10 (http://paup.csit.fsu.edu/about.html). Primers used for amplification of product for sequencing were 5′-AATCCAGATGTCATCAGGAAAC-3′ and 5′-CCTATAGATGTGAACACTCCTCC-3′. The E gene sequences were consolidated from overlapping, bidirectional sequences. Scale bar indicates nucleotide substitutions per site.

## Conclusions

Dengue has reemerged in Mauritius after >30 years, but the outbreak was short-lived because of the institution of control measures and the arrival of cooler and drier weather. In the affected areas, monthly mean maximum temperature dropped from 28.3°C in June to 26.4°C in August, and total monthly rainfall amount fell from 126.4 mm in May to 44.8 mm in August. The outbreak was also restricted to some suburbs of the capital city, possibly because of relatively warm temperatures and high population density. The reemergence was probably caused by introduction of DENV-2 by unrecognized infective travelers. The high bootstrap value of 94% in the phylogenetic analysis suggests at least 2 separate importations of DENV-2 occurred. In 2008, an imported case of dengue was diagnosed in a child returning from India, but control measures were rapidly instituted and no local transmission occurred.

No case of dengue hemorrhagic fever was recorded in this outbreak, probably because the population has not been exposed previously to another serotype. The vector of the outbreak was likely to have been *Ae. albopictus* mosquitoes, which are widely distributed in Mauritius (*Ae. aegypti* was eradicated from the country in the early 1950s as a result of a DDT indoor-spraying campaign in 1949–1951 to control malaria) (*7*). However, the rapid increase in the number of observed cases in June is more consistent with an *Ae. aegypti*–borne dengue outbreak, and a new comprehensive entomologic study is needed to exclude the possibility that *Ae. aegypti* has recently been reintroduced into Mauritius.

Whether DENV-2 will persist in Mauritius throughout the winter and lead to more cases next summer, despite maintenance of intensive mosquito control programs, is uncertain However, all practical measures must be taken to prevent introduction and transmission of another DENV serotype in Mauritius to minimize the risk for dengue hemorrhagic fever. In particular, surveillance of travelers from dengue-endemic regions should be instituted. The thermal scanner, recently installed at Mauritius’ only airport, could be used to screen passengers for dengue fever because a study from Taiwan suggested that fever screening at airports was a cost-effective means of identifying many imported dengue cases (*8*). Moreover, the present policy of monitoring all persons arriving from malaria-endemic areas for fever and parasitemia could be extended to include testing for dengue in febrile travelers arriving from dengue-endemic areas. The recently opened CHL molecular biology unit needs to be ready by next summer to detect and serotype dengue viruses to enable prompt diagnosis and epidemiologic evaluation of any new case.
